# Surgical Interventions for NEC—An Overview

**DOI:** 10.3390/children13070939

**Published:** 2026-07-17

**Authors:** Tirion Hughes, Naomi Wright, Kokila Lakhoo

**Affiliations:** 1 Medical Sciences Division, University of Oxford, Oxford OX3 9DU, UK; tirion.hughes@msd.ox.ac.uk; 2 Oxford University Hospitals NHS Foundation Trust, Oxford OX3 9DU, UK; naomi.wright@ouh.nhs.uk; 3 Nuffield Department of Surgical Sciences, Global Surgery, University of Oxford, Oxford OX3 9DU, UK

**Keywords:** necrotising enterocolitis, paediatric surgery, neonatal surgery, paediatric surgical outcomes, outcome reporting, neonatal laparotomy, neonatal intensive care unit

## Abstract

Necrotising enterocolitis is a highly challenging condition in neonatal surgery, with a substantial proportion of affected infants requiring operative intervention with a significant associated morbidity and mortality. Radiological evidence of perforation represents a clear indication for surgery; however, in many other cases, the disease presentation and progression are heterogenous, with significant diagnostic and prognostic uncertainty. The surgical assessment and decision making in these cases relies on integrated judgement of the clinical trajectory, examination, and biochemical and radiological findings, as well as consideration of the comorbidities and gestational age. Although many have been proposed in the literature, there remains to be any universal validated thresholds or criteria for reliably identifying those infants who may be ‘failing medical management’ and are likely to develop intestinal necrosis. Similarly, comparisons of different surgical approaches (stoma, primary anastomosis, and damage-control strategies) and outcome reporting are often confounded by the disease severity index and patient selection, as well as the generally observational nature of studies within the field. Improved standardisation of disease and severity definitions, and the development of validated prognostic tools, will facilitate the earlier identification of infants most likely to benefit from surgery, which in turn could reduce delays to theatre and improve the outcomes. Multi-centre collaborative datasets provide important evidence in the path to achieving this aim, by providing valuable cohort and outcome data, which also contribute to informed shared decision making with parents and families.

## 1. Introduction

NEC is a potentially disastrous illness in preterm infants with a significant mortality and morbidity, including long-term growth and neurodevelopmental impairment. Surgical NEC is associated with the most severe end of the disease spectrum, thereby contributing significantly to the long-term health burden associated with the condition. We aimed to review surgical interventions for NEC and the current best evidence to guide clinical practice and further research in the field.

## 2. Difficulties in Diagnosing NEC and Defining ‘Surgical NEC’

### 2.1. Defining Surgical NEC

Whilst many infants with necrotising enterocolitis (NEC) can be managed conservatively with medical intervention, around one quarter will require surgical intervention—27.9 per 100,000 live births [1]. National surveillance datasets play an important role in improving the understanding of surgical NEC, given the relative rarity at the individual-centre level and the heterogeny of the definition and severity reported in single-centre cohorts. BAPS-CASS (British Association of Paediatric Surgeons Congenital Anomalies Surveillance System) is a national surveillance platform that enables the prospective study of uncommon paediatric surgical conditions, including surgically managed NEC, across the UK. It was developed to address the limitations of single-centre series by allowing population-based data collection using structured reporting similar to other national rare disease surveillance systems. Multi-centre prospective cohort studies underpin the key evidence available in this area; randomised controlled trial (RCT) data are scarce due to the methodological, statistical, and potentially ethical difficulties with conducting a comparative study in a highly heterogenous and often critically unstable cohort, with an emergent surgical condition requiring urgent intervention. A summary of RCTs in the field of the surgical management of NEC, identified by a systematic literature search, is included in Section 4.

The BAPS-CASS study of surgical NEC was conducted over a 13-month period that aimed to capture all infants meeting the predefined criteria in participating centres. An analysis of these data has provided a valuable characterisation of the epidemiology, indications and surgical decision making, variations in management, and estimates of the mortality, morbidity, and long-term outcomes [1,2,3,4]. UK NEC outcomes are also monitored by Children’s Surgery Outcomes Reporting (CSOR), which allows for longitudinal auditing and cross-centre learning on several paediatric surgical conditions’ management and outcomes. Given the paucity of standardised definitions and comparative RCT data in the field, multi-centre prospective cohorts such the UK BAPS-CASS analyses assist in informing clinical decision making as well as advising discussions with parents and family about the risks and outcomes. However, these data must be interpreted with care given the confounding effects seen in observational studies.

Surgical NEC is classically defined within the published literature as NEC cases requiring surgical intervention; however, this definition may miss patients who do not survive to surgery or are redirected towards palliative care before surgery occurs. Cases of surgical NEC were identified in the BAPS-CASS cohort using combinations of the Vermont Oxford criteria and visualisation of the NEC, either at the time of surgery or post-mortem. Infants with NEC were included if they were deemed to require surgical intervention, regardless of whether surgery was actually performed. Due to the challenges in differentiating spontaneous focal intestinal perforation (SIP) from surgical NEC pre-operatively (especially when using the Vermont Oxford criteria), both were included in the BAPS-CASS cohort, with subgroup outcome analyses [1]. Although these pathologies both require surgical intervention, the details of the surgical management and outcomes are distinct.

Despite the relatively simple definition of surgical NEC, the main challenge arises when attempting to differentiate this from medical NEC or in clinical practice—in the decision to proceed (or not) to surgical management, often without definitive surgical indications.

### 2.2. Severity Scoring and Risk Stratification Models

The modified Bell’s staging criteria represent the most widely recognised and utilised clinical staging score in NEC, categorising the severity based on systemic, intestinal and radiological findings. They have been in use since the late 1970s, both clinically and within registries and datasets, and they range from suspected NEC to overt perforation. They were originally proposed by Bell in 1978 and modified subsequently with substages, as described in Table 1 [5,6]. Although Bell’s criteria are still widely used to classify the disease severity in NEC, both clinically and academically, they are limited in their utility for predicting surgical disease or even reliably identifying NEC. Many of the early features in stage I and II are non-specific and could result in a misdiagnosis or the delayed diagnosis of another condition such as non-abdominal neonatal sepsis, milk curd obstruction or intestinal malrotation with volvulus. Notably, the modified Bell’s criteria are not able to reliably differentiate NEC from SIP. Furthermore, several risk-stratifying factors important for surgical decision making are not considered within Bell’s, including comorbidities and the gestational age. Due to these limitations, when used in isolation, it has a limited utility for identifying which infants will require surgical intervention.

A lack of clarity regarding the best practice for identifying surgical NEC has been a feature in the literature within the field for many years, and is a common concern of clinicians [7]. From a research perspective, inconsistencies between classification systems and definitions can impair the cross-comparison and centralisation of data from NEC patients in different studies [8,9]. Other scores and criteria have been proposed to improve the classification and diagnosis of NEC, as well as the early identification of infants with surgical NEC, including the UKNC-NEC gestational-age-specific definition, the CDC definition, the two of three rule, the INC NEC definition, the Stanford NEC score and the Vermont Oxford definition. A recent systematic review identified 63 unique reported methods designed to distinguish surgical from medical NEC, including composite clinical scores such as the metabolic derangement 7 (MD7) score, single biomarkers and imaging (primarily ultrasound-based) assessments [3]. Many early trials continue to investigate various potential biomarkers [10]. While several models demonstrate a moderate predictive performance, most have been developed in single-centre or small multi-centre cohorts, and few have undergone any robust external validation. Large multi-centre cohorts provide the opportunity to validate the most promising candidates, which include the MD7 score, plasma CRP trajectory, and abdominal US features (portal venous gas and fixed bowel loops). A recent RCT demonstrated the superiority of the combination of MD7 and paracentesis over Bell’s criteria in guiding surgical decision making, in terms of the subsequent re-operation rate and mortality [11]. However, no method yet predominates. Current practice continues to rely on a combination of clinical judgement and a composite interpretation of the disease trajectory, laboratory findings and imaging features, rather than a single standardised and validated prognostic tool. Emerging approaches should also aim to differentiate SIP from surgical NEC pre-operatively, to allow for better-informed surgical decision making, taking into account the difference in surgical approaches and prognoses between the two conditions. Artificial intelligence (AI) models may also play a role in the future, but require large-scale data for reliability, which is a challenge.

## 3. Surgical Interventions for NEC

There are several approaches to the surgical management of NEC, including primary peritoneal drainage and a laparotomy. During a laparotomy, potential interventions include stoma formation, primary anastomosis, and damage control approaches such as ‘clip and drop’, alongside various established and emerging adjunctive techniques. Selecting the appropriate interventions for an infant, as well as the timing and location of surgery, depends largely on the indication and assessment of the overall illness severity, alongside the extent of the disease identified intra-operatively.

### 3.1. Indications for Surgical Intervention

The widely accepted absolute indication for the surgical management of NEC is intestinal perforation, evidenced by pneumoperitoneum on an abdominal or chest X-ray, with intestinal perforation being the most frequent indication for surgery in the BAPS-CASS cohort (50.4%, 67/133) [1]. Although pneumoperitoneum is a strongly specific finding indicating intestinal perforation, this encompasses both NEC and SIP and does not differentiate between the two. In a Saudi Arabian multi-centre cohort, pre-operative pneumoperitoneum was present in 53% of infants found to have NEC at the time of operation, but was present in 100% of those found to have SIP [12]. Radiological pneumoperitoneum also has a low sensitivity, being present only in a proportion of infants requiring surgery for NEC. In a small Spanish single-centre cohort, only 36% of the infants found to have intestinal perforation at the time of the laparotomy had pre-operative pneumoperitoneum, with a small UK cohort of infants undergoing surgery in the NICU for demonstrating a similar figure of 33% [13,14].

In the absence of pneumoperitoneum, the decision to operate relies on relative indications that attempt to differentiate infants with evolving intestinal necrosis or softer signs of perforation from those who can continue to be managed medically. These features include a variety of examination findings (abdominal tenderness, palpable mass, abdominal wall oedema/erythema) and radiological features (portal venous gas, pneumatosis, ascites, fixed bowel loop). These are taken together with systemic signs (frequency of bradycardias/apnoeas, inotrope requirement) and laboratory values (CRP, acidosis) to establish a picture of the overall clinical trajectory and support escalation to surgery in cases where the risk of perforation or necrosis is significant.

Clinical uncertainty regarding surgical decision making is naturally heightened in non-pneumoperitoneum cases, which in turn can lead to a longer time to theatre. An analysis of the BAPS-CASS dataset demonstrated that, for infants with ‘failed medical management’ (comprising around a third of the cohort), the time to theatre was an average of 30 h longer, compared with those suspected to have perforation [3]. ‘Failed medical management’ as the indication for surgery is also a strong predictor of poor outcomes. However, there is no universal definition of failure of medical management, further contributing to uncertainty and inconsistency in decision making [7]. Recognising which infants are failing medical management with enough time to pre-empt perforation or catastrophic progression is a key challenge within the field.

### 3.2. Timing of Surgical Intervention

The paucity of clearly defined criteria for surgical intervention in NEC (beyond features of perforation), as well as potentially confounding differential diagnoses, means that surgical timing and urgency is based primarily on individual clinician judgement. This judgement uses pattern recognition of a variety of the clinical and radiological features described above, but without objective thresholds. For infants with non-specific signs, there exists a dilemma between early intervention to limit the spread of suspected intestinal necrosis and delayed surgery to allow for possible clinical improvement—potentially avoiding a laparotomy and an unnecessary loss of bowel length. Although preventing an unnecessary laparotomy is of course important, the outcomes in NEC are inextricably linked to the early diagnosis and identification of infants with surgical disease, emphasising the importance of optimising early identification as an opportunity to improve the outcomes.

### 3.3. Types of Surgical Intervention

#### 3.3.1. Peritoneal Drainage

**Figure 1:**
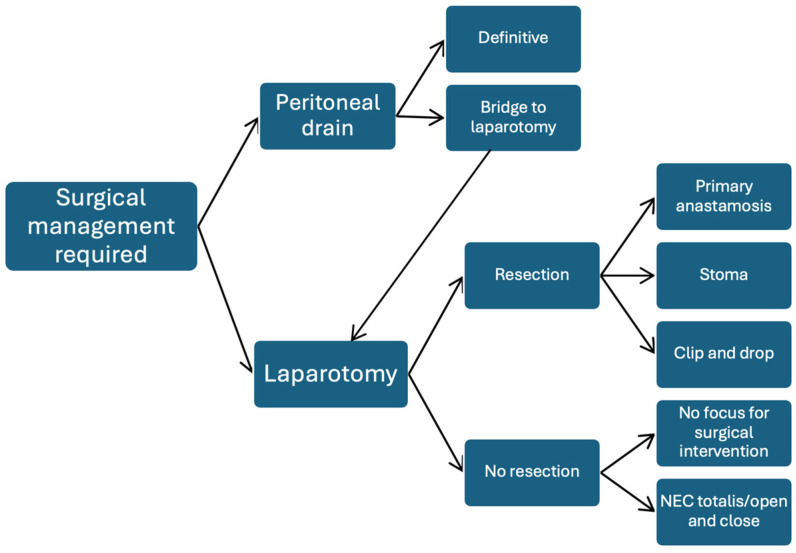
Operative strategies.

Once an infant has been identified as requiring surgical management for NEC, the first major decision is that of the surgical approach (Figure 1). Most infants will have a primary laparotomy, but other approaches are also used. Peritoneal drainage has been used historically, both as a temporising measure and with the aim of definitive management. This can be undertaken more readily at the bedside in the NICU, so it was traditionally used in those highly unstable infants deemed not stable enough for transfer or a laparotomy. RCT data have not demonstrated superiority of a primary drain insertion as definitive management over a laparotomy, with most studies demonstrating no improved outcomes with drain insertion, but a high likelihood of a subsequent laparotomy—demonstrated in a 2025 Cochrane Review [15,16,17]. In a 2008 RCT, 74% of infants randomised to receive drain insertion later required a laparotomy [18]. In the BAPS-CASS dataset, 7.5% of infants had drainage as the first intervention, but 100% of these went on to have a laparotomy [2,3]. Primary peritoneal drainage in place of a laparotomy is therefore not supported, but drainage as a bridge to definitive surgical management continues to be utilised in some settings, in critically ill infants such as those with abdominal distension impairing ventilation, or where there may be a time delay to laparotomy due to factors such as transfer to another centre [19]. However, RCT data suggest that, even as a temporising measure, drain insertion does not result in the post-operative stabilisation of very-low-birth-weight (VLBW) infants, so this remains an area of uncertainty and would benefit from further study of the long-term outcomes [20].

#### 3.3.2. Resection, Stoma Formation and Primary Anastomosis

The operative aim in a surgical NEC laparotomy is to resect necrotic or perforated bowel, and provide source control for sepsis, while preserving as much intestinal length as possible. In some cases, where an infant is very unstable or has multifocal patchy disease, diversion without resection may be utilised as a damage control strategy, to defunction the distal bowel and potentially avoid the extensive loss of intestinal length. However, in most cases, a focal area or areas of necrosis or perforation will be resected, usually alongside stoma formation. The convention for enterostomy over primary anastomosis is rooted in concern over an anastomotic leak, which is more likely when the bowel is poorly perfused, oedematous, or closely opposed to inflammation. It is therefore reasonable that many infants with NEC would not be appropriate for primary anastomosis, due to frank intrabdominal sepsis. Stoma formation also has the advantage of allowing the distal bowel rest, but it does come with the risk of fluid and electrolyte losses, delayed growth, a requirement for ongoing parenteral nutrition (PN), and the need for re-operation for closure. For these reasons, in recent years, there has been a shift towards primary anastomosis in selected cases, although in cases of extreme prematurity/an extremely low birth weight (ELBW), this can be particularly high-risk.

In the BAPS-CASS NEC cohort from the mid-2010s, stoma formation predominated, with 72% of the infants included having stoma formation (compared to 17% undergoing primary anastomosis) [1]. Retrospective studies have demonstrated that infants who undergo primary anastomosis have no significant mortality differences compared with those who undergo stoma formation, but may have shorter hospital stays and faster progression to feed [21]. However, the nature of observational outcome data in this area can be challenging to interpret, as infants who are more unwell pre-operatively are more likely to undergo a stoma than anastomosis, which can confound the outcome comparisons. In addition, infants with SIP are often not excluded in these cohorts. Still, there is higher-order evidence in support of primary anastomosis in selected cases—the 2024 STAT trial, a worldwide multi-centre RCT, found that primary anastomosis resulted in no difference in mortality or adverse outcomes, but the rate of multiple intestinal complications was increased in the stoma group, and infants were quicker to finish parental nutrition in the primary anastomosis group. The STAT trial discussion concluded that, where there is no disease distal to the site of resection, recovery is enhanced by opting for primary anastomosis [22]. Primary anastomosis versus stoma formation in an emergency laparotomy is a recurring theme in abdominal surgery, beyond NEC (and beyond paediatric surgery), with the general trend moving further in favour of primary anastomosis where appropriate. Individual surgical judgement of the bowel at the time of surgery remains central for identifying those infants who are unsuitable for primary anastomosis, with particularly careful consideration in infants of extreme prematurity, in whom anastomosis carries a higher risk.

#### 3.3.3. Damage Control Surgery and Pan-Intestinal Disease

Some of the most challenging operative scenarios are those where the infant is critically unwell, and where NEC is multifocal or approaches pan-intestinal. For profoundly unstable infants who may not tolerate a prolonged laparotomy, damage-control strategies are a recognised technique to prioritise rapid stabilisation over definitive surgical management. This can include ‘clip and drop’ (whereby clearly necrotic or perforated segments are resected, but moderately affected areas are left in situ, and the intestinal ends are clipped or tied and remain intra-abdominal) or limited resection/diversion such as a high diverting jejunostomy, with a planned second-look laparotomy for a staged reassessment of the borderline salvageable bowel once the physiology is better stabilised. Naturally, enteral feeding is not an option in this interim period, prolonging the reliance on PN. A total of 5% of the BAPS-CASS NEC cohort had ‘clip and drop’ [1]. Peritoneal lavage may be used as an adjunct to damage control surgery or drain insertion in cases of perforation or diffuse peritonitis. Historical publications have also explored post-operative continuous peritoneal lavage, based on the principle of reducing intrabdominal contamination and proinflammatory mediators; however, this effect is largely theoretical and not explored in recent studies [23].

A 2025 prospective cohort study demonstrated better outcomes than historical datasets when employing a ‘damage control surgery’ approach for a select group of seriously ill infants with perforation and/or requiring inotropes [24]. However, as studies of this critically unwell cohort are limited by an unavoidable selection bias, the field lacks comparative data and robust outcome reporting. Decision making in infants with pan-intestinal NEC must be centred on balancing survival against lifelong short bowel syndrome, which is associated with significant morbidity and mortality.

Some infants found to have pan-intestinal NEC intra-operatively are considered to have ‘NEC totalis’, where >75% of the intestinal length is affected. In these cases, there may be no clear demarcation of salvageable/necrotic bowel, and/or only a very short segment of salvageable bowel present. If there is no meaningful length of viable bowel, care will likely be redirected towards palliation (often termed an ‘open and close’ laparotomy). NEC totalis resulting in an ‘open and close’ laparotomy was the outcome in 5% of the BAPS-CASS cohort, so the incidence is likely to be at least 1 in 20 of surgical NEC cases [1].

#### 3.3.4. Laparoscopy

A laparoscopy has also been explored as an alternative operative approach in selected infants with NEC, primarily as a means of improving the diagnostic accuracy and limiting unnecessary laparotomies. It can allow for the direct visualisation of the bowel to assess the viability, the extent of necrosis, and the presence of perforation, and in some cases, may prevent a further laparotomy if the findings are reassuring. However, a laparoscopy in VLBW infants can be limited by technical challenges at this low body weight, as well as intra-operative physiological instability due to maintaining iatrogenic pneumoperitoneum. A dilated bowel can also limit visibility, and hence, utility. Most evidence in this area is in the form of small case series and retrospective cohorts, and there are no RCTs. It remains a diagnostic tool only, with utility in a select group of infants for whom there is significant uncertainty in the diagnosis or severity, and who are also able to tolerate a laparoscopy [25,26].

#### 3.3.5. Adjuvant Surgical Techniques

Indocyanine green fluorescence imaging (ICG-FI) is an emerging adjunct in the management of surgical NEC, particularly in assessing bowel viability during second-look surgery. ICG-FI involves the intra-operative intravenous administration of ICG, which emits near-infrared fluorescence, enabling the real-time assessment of bowel perfusion as a surrogate marker of viability. Similar ICG-FI techniques are already utilised in various paediatric hepatobiliary, cardiothoracic and oncology surgeries. Recent observational work in neonates with extensive NEC undergoing a second-look laparotomy suggests that the absence of fluorescence corresponds with histological necrosis, even where this differs from conventional macroscopic assessments [27]. An earlier study proposed the use of ICG-FI in combination with a laparoscopy to aid in the early identification of necrosis [28]. An experimental animal model of NEC has also demonstrated the potential for quantitative ICG-FI to distinguish necrotic from salvageable bowel [29]. Wider use within the field of NEC will require standardisation, including dosing protocols and the quantification of fluorescence interpretation. The feasibility of intra-operative quantitative ICG-FI to identify bowel perfusion patterns has recently been demonstrated in a cohort of adult colorectal cancer patients [30]. Other methods of the intraoperative assessment of intestinal perfusion are emerging, including laser speckle contrast imaging (LSCI), a dye-free optical technique that has been demonstrated in adult colorectal surgery cohorts [31]. A pre-clinical mouse model of NEC supports the potential for LSCI use in assessing intestinal microcirculation; however, the evidence of clinical utilisation in NEC surgery is limited to the single-case-report level [32,33].

### 3.4. Surgical Setting

Many infants are born in neonatal units without on-site paediatric surgery, requiring transfer to tertiary centres prior to the laparotomy. Of the BAPS-CASS cohort, 70–78% (depending on the indication for a laparotomy) were transferred from another centre for surgical care [3]. Although it may be expected that transfer increases the morbidity, this has not been demonstrated in the literature. An analysis of the BAPS-CASS outcomes found that pre-operative transfer from another NICU was associated with an increased survival and reduced PN requirement at 28 days. However, as ELBW neonates with a poorer prognosis are more likely to have already been directed to specialist centres, this likely represents the lower gestational age/weight of the cohort that did not require transfer, rather than any direct effect. A recent metanalysis investigating the association between surgical transfer (for any indication) and the mortality in neonates in high-income countries also did not show any increased mortality or long-term disability associated with transfer for surgical intervention, including in a specific subgroup analysis of NEC, although infants deemed unsuitable for transport from their initial centre were not included [34].

Selecting the optimal surgical setting within a centre for operating on infants with NEC is a complex decision reflecting a balance between the clear advantages of the operating theatre environment against the risk of transferring an unwell neonate. It is also heavily influenced by centre-specific factors. Where feasible, the operating theatre is clearly the ideal location—offering better lighting, instrument availability, and familiarity for surgical and anaesthetic teams. However, a substantial proportion of infants with surgical NEC are physiologically unstable, often requiring inotropic support, high-frequency oscillatory ventilation, and central vascular access. Hence, the risks of transfer include the worsening of already fragile pre-operative cardiorespiratory instability, hypothermia, and the displacement of endotracheal tubes and other critical tubes/lines, as well as the delays associated with transferring a critically unwell neonate. As an alternative, a bedside laparotomy in the NICU was demonstrated as a feasible alternative in the early literature within the field [35,36]. However, operating in the NICU environment is not risk-free either, with the potential for a less-comprehensive assessment of the disease extent and the viability of borderline areas of the bowel, due to factors such as lighting and positioning. Concerns about sterility and the associated infection risk are well recognised, although a recent systematic review of NICU operations for multiple neonatal surgical conditions did not demonstrate any increase in post-operative wound infections in NICU-operated infants compared with those taken to operating theatres, although NEC-specific outcomes were not reported separately [37].

Contemporary case series and observational studies confirm the feasibility, both specifically for NEC laparotomies and in wider paediatric surgery. In several 10-year retrospective case series comparing NICU and operating theatre surgeries, no difference in the rate of surgical adverse events could be attributed to the surgical setting (e.g., surgical field contamination, lack of essential equipment availability) [13,38]. Some publications within the field have demonstrated a higher mortality in NICU-operated infants compared with the operating theatre; however, there is strong confounding by pre-operative illness severity [13]. In the majority of centres, the risks of transfer would only outweigh the risks associated with a bedside laparotomy for those most critically unstable and/or ELBW infants. Studies based in centres with lower thresholds for bedside operations (such as those who routinely operate bedside for all infants below a certain birth weight or any intubated neonate) usually demonstrate a lower mortality, which trends towards the expected mortality for the surgical indication [36,38]. Conversely, those with a very high threshold for bedside surgery (such as inotrope or high-frequency ventilation) demonstrate a much higher mortality, including a higher-than-usual proportion of open-and-close cases and deaths in the immediate post-operative period [13]. Taken together, these publications support the view that bedside surgery is a safe and feasible alternative for critically ill neonates, and that the pre-operative comorbid status is the main factor affecting the observed mortality differences, rather than the surgical setting. This conclusion is also reflected in metanalyses of bedside neonatal surgery [37,39]. Additional benefits associated with avoiding transfer are harder to quantify.

Patient selection for a bedside laparotomy remains non-standardised. Most centres do not have formal institutional guidelines for bedside surgery—guidelines were reported in 33% of centres in a recent systematic review [39]. There is no prediction model that has been validated to identify those infants for whom the risk of transfer outweighs the risk associated with a bedside laparotomy [39]. The centre configuration significantly influences the choice of surgical setting; naturally, those centres with a very long distance between the NICU and the operating theatres will likely have a lower threshold for avoiding the transfer of unwell neonates, whereas those with closely apposed theatres/NICU may consider the transfer of more unstable infants. As described above, mortality differences between bedside and theatre laparotomies are therefore likely to vary between centres and will in part correspond with the local clinical thresholds and criteria for bedside laparotomies. Overall, a bedside laparotomy in the NICU represents an established strategy for managing the risk of transferring critically ill neonates with surgical NEC. However, given the paucity of prospective and RCT-level evidence, care should be taken to assess the risk and prioritise a bedside laparotomy only for very unstable infants, or in centres where transfer is associated with higher risks due to institutional factors. Centres performing a bedside laparotomy should ensure the streamlining of systems to ensure the fast, but thorough, set-up of operative equipment and staff in the NICU, as well as early tripartite decision making between paediatric surgeons, paediatric anaesthetists, and neonatologists.

### 3.5. Post-Operative Course and Outcomes

#### 3.5.1. Predicting Mortality

Surgical NEC remains associated with a substantial short- and long-term morbidity and mortality. These outcomes are heavily influenced by the gestational age, birth weight, physiological instability/comorbidities, and distribution of disease. Those infants requiring surgical management for NEC experience a higher mortality and an increased risk of significant morbidity, such as a growth impairment, PN requirement or neurodevelopmental impairment, compared with NEC managed exclusively medically, reflecting the greater severity of disease [2,40]. Of the BAPS-CASS cohort, 15% of those found to have NEC at a laparotomy had died at the 28-day follow-up, rising to 22–38% in the same cohort at 1 year [1,2].

A number of factors, beyond surgical disease, are associated with an increased mortality. These include demographic factors—gestational age and a small-for-age weight are both unsurprisingly correlated with a greater mortality. For the most extreme low-birth-weight infants requiring surgery for NEC, the overall mortality rises to 50% [41]. Comorbidities and indicators of a poor physiological baseline are associated with an increased mortality, including congenital abnormalities, and the requirement for ventilation or inotropes at the time of presentation [3]. Non-disease-specific scores such as the Paediatric Index of Mortality (PIM2) have not demonstrated utility when applied in an attempt to identify those infants that will have poor outcomes after a laparotomy [13].

Infants for whom the indication for surgery was ‘failed medical management’ (rather than concern for perforation or necrosis) suffer worse outcomes, with more than four times the odds of death or PN requirement at 28 days [3]. A prolonged time to theatre is likely to be a contributory factor; hence, improving the early recognition of infants who will require surgical management of NEC (in the absence of absolute surgical signs) is an area of opportunity for reducing mortality. The disease extent is of course a significant factor—with multifocal disease associated with poorer outcomes and the presence of any length of normal bowel being a protective factor. In terms of identifying infants with high-risk surgical disease, abdominal wall erythema/discolouration was a stronger predictor of 28-day mortality than radiological evidence of perforation within the BAPS-CASS cohort [1].

The necessity for clip-and-drop surgery is associated with a significantly higher early mortality, reflective of the greater disease severity at index operation [1]. Comparative data between operative strategies remain limited due to confounding by indication, but the recent STAT trial suggests a comparable survival between those infants who undergo stoma formation and primary anastomosis, where these are appropriately selected for the extent and spread of the disease seen [22].

#### 3.5.2. Surgical Complications in NEC and Re-Operation

There are a number of well-recognised complications of surgical NEC, with most being sequelae of intestinal inflammation, ischaemic injury and necrosis, as well as intra-abdominal sepsis. Many of these require further surgical management—two thirds of infants with surgical NEC will require at least one further surgery within the first year [2]. A recent multi-centre retrospective cohort study of neonates undergoing surgery for NEC demonstrated a 30-day complication rate of 57%, the majority of which could be attributed either to sepsis, wound dehiscence, or stomas [42]. Similar factors to those affecting mortality were found to increase the risk of complications, including the inotrope requirement pre-operatively.

Post-NEC intestinal stricture is a frequently described complication of NEC and often requires an additional surgery for resection and anastomosis of the affected site. They are usually diagnosed by a contrast study, and associated with a significant ongoing morbidity due to intestinal obstruction. Stricture is common in infants having undergone medical management of NEC, due to intestinal inflammation (17% of a retrospective NEC cohort) [43]. However, the risk is higher after surgical management (24%), presumably due to an increased severity of ischaemic changes present in the surgical group [43]. Beyond the surgical status, other factors are associated with an increased stricture risk, including high peak CRP during their initial disease course, with a large retrospective cohort study not identifying any post-NEC stricture patients who had a CRP level < 46 mg/L [43]. An important differential in cases of post-NEC intestinal obstruction is adhesional bowel obstruction, which may present similarly to stricture and can be investigated by a contrast study (likely to demonstrate a transition point, rather than tapering) or a diagnostic laparotomy. In the early post-operative period, the recurrence of NEC should also be a key differential, occurring in 5–8% of cases [44].

The predominant long-term complications of surgical NEC are short bowel syndrome and intestinal failure, seen in patients with limited residual bowel—subject to both the total residual length and the loss of specific areas of the bowel such as the ileocaecal valve, which is a poor predictor of later intestinal function. These are associated with a significant morbidity and mortality, and in cases requiring intestinal transplantation or long-term PN, which is associated with significant hepatotoxicity, the outcomes are particularly poor. Ongoing growth failure is a concern identified in both observational and RCT cohorts [40,45]. Post-surgical nutrition is an important factor to consider; a recent joint position paper encourages a general approach of 5–7 days of post-operative parental nutrition to allow for intestinal rest, followed by the slow introduction of enteral feeding, with human milk being the preferred option where available [46]. However, many infants require a prolonged period of PN post-operatively, indicating ongoing intestinal dysfunction. This is predictive of the longer-term outcome—infants with a PN requirement at 28 days had a nearly four-times-increased odds ratio for 1-year mortality in the BAPS-CASS cohort [2]. Data regarding the prevalence of intestinal failure following NEC are limited due to the observational nature of most studies and discrepancies in classification, but a large retrospective metanalysis estimated a 22–35% risk of intestinal failure in surgically managed infants (substantially higher than those managed medically) [41]. Nonetheless, a follow-up study of a wide cohort of children with intestinal failure suggested that those with post-NEC intestinal failure (which accounted for 34% of the cohort) are more likely to achieve enteral autonomy (not reliant on PN) even after prolonged PN exposure, compared with other causes of paediatric intestinal failure [47]. A larger cohort study of ‘severe surgical NEC’ (<30 cm residual bowel) found that, of the survivors, 32% were eventually weaned from PN [48]. This demonstrates potential for a positive prognosis in a small proportion of very severely affected infants, if they survive to hospital discharge. As a general guide for predicting the future weaning ability, those infants with >25 cm of residual bowel with a retained ileocaecal valve, or >40 cm without a retained ileocaecal valve, have the potential to come off PN.

#### 3.5.3. Stoma Complications and Timing of Closure

Stoma-related morbidity is common and usually occurs in the early post-operative period—as the aim in most cases is to reverse the enterostomy later in the clinical course. Most stoma-related complications can be classified as either structural issues with the stoma (prolapse, retraction, necrosis, stenosis, parastomal hernia, skin breakdown) or secondary effects, such as an electrolyte imbalance or growth impairment due to high outputs. The latter will depend greatly upon how proximal the stoma is, with transverse/descending colon stomas being less likely to have a clinically significant high output. Growth during the period with the stoma in situ is commonly impaired for infants post-NEC due to the high output and the sodium and fluid depletion.

In a small cohort of surgical NEC patients, 89% had weight-for-age deterioration in the period prior to closure, and 42% met the WHO criteria for ‘severely underweight’ [49]. Where these effects are severe, for instance, very high outputs requiring IV maintenance, a reduced enteral feed volume and ongoing PN, early closure may be warranted. However, there is limited comparative data evaluating the optimal timing for closure. Most evidence comes from a systematic review of observational data, which are generally supportive of avoiding a prolonged delay to closure. Multiple recent single- and multi-centre cohort studies have not found a significantly different post-closure complication rate between the early and delayed closure groups following an enterostomy for NEC, nor any significant disparity in the readmissions, length of stay, mortality, or reoperation [50,51]. Early closure groups did experience faster gastrointestinal recovery (time to first defecation/flatus) and a trend towards a higher weight at 6 months CGA [50]. Taken together, these findings would support early closure where feasible, which could be as early as a few weeks after the index procedure if high outputs necessitate ongoing IV replacement of losses. Multi-centre randomised trials comparing early vs. late stoma closure in post-surgical NEC patients would be beneficial in furthering higher-order evidence within the field.

The enteral intake should be increased only until the threshold of 20 mls/kg/day of stoma losses is met (above which is deemed ‘high output’). Increasing the enteral intake despite a high output results in excessive sodium depletion and an inability to grow. Weekly measurement of urinary sodium can help to identify infants with an overall body depletion of sodium (which may not show in serum sodium measurements). A urinary sodium < 20 often results in poor growth and should be treated, ideally with enteral sodium replacement where feasible, or IV if not. Typically, those with a distal ileostomy or colostomy with minimal small bowel loss will be more likely to tolerate full enteral intake without excessive stoma losses. Neonates with more proximal stomas have a greater likelihood of a high output and require PN supplementation until stoma closure. In some cases, substituting part of the milk intake with hydrolysed formula (alongside breastmilk) may help to increase absorption and reduce excessive stoma output whilst awaiting stoma closure.

Of note, the recent STAT trial (comparing primary anastomosis with stoma formation in selected patients) found that, although the rate of all complications was higher in the stoma group, these were almost exclusively stoma-related problems (high output, retraction, prolapse, etc.), and the rate of non-specific complications (stricture, wound infection, incisional hernia, wound dehiscence) was similar between groups [22]. The rate of multiple complications was higher in the stoma group, despite randomisation.

#### 3.5.4. Neurodevelopmental Outcomes

Emerging evidence over the past decades suggests a link between neonatal NEC and adverse neurodevelopmental outcomes, in a cohort already at a high risk due to non-surgical factors, including hypoxic ischaemic encephalopathy, congenital anomalies and other sequelae of extreme prematurity. Of children who experience NEC in the neonatal period, 25–60% are reported to have a long-term neurodevelopmental disability [41]. In terms of surgical NEC as a subgroup, a prospective cohort study demonstrated neurodisability to be more common in a surgical NEC cohort than a medical one, with 38% demonstrating a severe neurodisability at 18–24 months, compared to 24% in the medical NEC group and 17% in a VLBW non-NEC group [52]. Although this represents a composite outcome influenced by prematurity, systemic illness and intensive care exposure, surgical NEC is consistently identified as a subgroup with a particularly high ongoing burden of health needs, reflecting both the disease severity and the cumulative impact of multiple early adverse events. As such, surgical NEC functions not only as a surgical diagnosis, but also as a marker of increased long-term neurodevelopmental vulnerability and complex healthcare needs. In terms of improving the outcomes, future work may aim to identify if any subpopulations of infants within the surgical NEC population (for instance, stratification by birth weight or comorbidities) are at a higher risk for adverse neurodevelopmental outcomes, and may benefit from earlier surgical intervention or a particular surgical approach.

## 4. Summary of Randomised Controlled Trials of Surgical Interventions in NEC

A systematic search of three databases (Medline, Cochrane, and Embase) for RCTs published since the year 2000 concerning the surgical management of NEC was undertaken using the search strategies in Appendix A. This identified 30 unique publications. A total of 22 were excluded due to: duplication (*n* = 1), not being specific to NEC (*n* = 7), not being specific to surgical management (*n* = 8), and not an RCT (*n* = 6). Trials including NEC patients alongside other pathologies such as SIP were included only where the NEC subgroup outcomes were reported separately. This identified eight RCTs within the field of surgical NEC management published since 2000. Five of these trials are summarised in Table 2—four relate to the surgical approach (three comparing a laparotomy with a peritoneal drain and one comparing primary anastomosis with stoma formation), while the final trial compares surgical decision-making strategies. A further three were not separate RCTs, but planned secondary studies or post hoc analyses of the NEST trial, and are therefore not included in the main body of the table.

## 5. Discussion

Surgical NEC represents a challenging area of neonatal surgery, not only because of the severity of intestinal injury that may be encountered, but because decision making frequently occurs in the setting of diagnostic uncertainty, rapidly evolving physiological instability, and a profound underlying vulnerability related to extreme prematurity and/or comorbidities. Although perforation remains the clearest indication for operative intervention, much of the complexity in contemporary practice lies in identifying surgical disease in infants with more insidious and often subtle deterioration before catastrophic deterioration occurs. Deterioration may occur over hours to days rather than minutes, yet progression to transmural necrosis or perforation can be rapid and unpredictable. Surgical decision making and urgency therefore often reflect the overall trajectory rather than a single clinical threshold.

Despite advances in neonatal care, surgical NEC continues to be associated with a substantial mortality and long-term morbidity. Decisions regarding the laparotomy, the extent of resection, the anticipated bowel function, the potential for PN dependence and further surgical procedures, and in some cases, redirection towards palliative care, often occur in the context of rapidly evolving deterioration in extremely preterm infants with multiple comorbidities, with parents who have had little time to come to terms with these challenges. The qualitative BAPS-CASS sub-study exploring clinician perspectives on decision making in severe NEC highlighted the complexity of balancing survival against the potential for severe intestinal failure and other impairment, particularly in cases of multifocal disease or NEC totalis [7]. Clinicians described prognostic uncertainty, variability in individual thresholds for intervention, and the challenge of communicating risk to parents in time-critical situations. Multidisciplinary collaboration between neonatologists, paediatric surgeons, NICU nursing teams and other specialists can support families in navigating these complex decisions; studies examining the parental experience emphasise clear communication, consistent messaging and acknowledgement of uncertainty.

Infants requiring operative management represent the most severely affected subgroup of the NEC population, and the outcomes reflect the cumulative burden of prematurity, systemic inflammation, and intestinal injury. Stoma-related morbidity and the need for further operations are common in the early post-operative course, whereas short bowel syndrome; the sequelae of long-term PN, including hepatic dysfunction; and neurodevelopmental impairment remain major contributors to the long-term disease burden. Impaired growth is a concern throughout, particularly in infants affected by high-output stomas or short bowel syndrome. Importantly, these outcomes are not solely post-operative complications, but are heavily influenced by the extent of the original disease and the presence of comorbidities of early prematurity. Despite the feasibility and acceptability of multiple surgical approaches, a recurring theme throughout the literature is the difficulty of comparing outcomes between operative strategies due to the confounding by disease severity. Infants selected for stoma formation, damage-control surgery or a bedside laparotomy are often those with the greatest physiological compromise or most extensive intestinal disease, complicating the interpretation of the observational data. While randomised evidence is beginning to emerge in selected areas, the evidence base remains primarily derived from multi-centre observational cohort studies and national surveillance datasets such as BAPS-CASS, which have made important contributions by helping to contextualise findings from smaller single-centre series and identify priorities for future research.

Future progress in surgical NEC is likely to depend on improving the identification of infants with evolving surgical disease, and better prognostication pre-operatively to identify which infants are most likely to benefit from bowel-preserving approaches (and for which infants there is little chance of a positive outcome). The absence of validated objective thresholds in these areas represents a major limitation within the field, contributing to substantial variation in the thresholds for surgical referral and intervention between centres, particularly in infants without pneumoperitoneum. Future research priorities should therefore include the validation of prognostic tools capable of distinguishing surgical from medical disease earlier in the disease course, alongside improving the standardisation of definitions and outcomes to enable meaningful comparisons between studies, and continued collaboration through national/international surveillance networks.

## Figures and Tables

**Table 1:** Modified Bell’s criteria.

Stage	Abdominal	Radiographic	Systemic
Ia/Ib	Increased gastric residuals, vomiting, mild distension, occult blood in stool (if frank PR blood, Ib)	Mild ileus or normal	Apnoeas, bradycardia, temperature instability
IIa (mild)	Ia/b plus reduced bowel sounds, possible abdominal tenderness	Ileus, dilatation, pneumatosis	As in Ia/b
IIb (moderate)	IIa plus definite tenderness and abdominal wall oedema, possible palpable mass	IIa, but more extensive pneumatosis, possible ascites and portal venous gas	Ia/b plus thrombocytopaenia and mild acidosis
IIIa (advanced)	IIb with worsening wall oedema and erythema	IIb, but with prominent ascites, static bowel loop	Evidence of end organ disruption, worsening acidosis, hypotension, oliguria, DIC
IIIb (advanced)	Clinical signs of perforation	Pneumoperitoneum	Worsening of the above, evidence of shock

**Table 2:** Randomised controlled trials in surgical management of NEC, 2000–2026.

	Title	Description	Summary of Findings
PMID: 16723614 [16] Moss et al. 2006	Laparotomy versus peritoneal drainage for necrotizing enterocolitis and perforation	Multi-centre RCT comparing primary laparotomy and resection with primary peritoneal drainage in perforated NEC. A total of 117 patients randomised. No statistically significant differences in terms of survival at 90 days, PN dependence at 90 days, or length of stay. No significant advantage in any subgroup analyses, including subgroups of <25 weeks, presence/absence of radiological evidence, or pH < 7.3.	Primary laparotomy versus peritoneal drainage for perforated NEC does not influence survival or other early outcomes.
PMID: 20152345 [18,20] Rees et al. 2008, 2010	Peritoneal drainage does not stabilize ELBW infants with perforated bowel: data from the NET Trial	Multi-centre RCT comparing primary laparotomy with primary peritoneal drainage in ELBW infants with pneumoperitoneum. A total of 69 patients randomised to PD (*n* = 35) or LAP (*n* = 34). No post-procedure improvement in either group when comparing heart rate, BP, inotrope requirement or PaO_2_/FiO_2_. Infants managed with PD had a poorer post-op cardiovascular status. No differences in total organ failure score in either group.	Peritoneal drainage does not immediately improve clinical status in ELBW infants with bowel perforation (combined NEC and SIP population)
PMID: 34506326 [17] Blakely et al. 2021	Initial Laparotomy Versus Peritoneal Drainage in ELBW Infants With Surgical Necrotizing Enterocolitis or Isolated Intestinal Perforation: A Multicentre Randomised Controlled Trial (NEST) *	Multi-centre RCT comparing primary laparotomy versus peritoneal drainage, on rate of death or neurodevelopmental impairment (NDI) in NEC or SIP patients. A total of 310 randomised (95 of which were NEC). No overall difference in death or NDI rates between groups combining NEC and SIP. Preoperative diagnosis (NEC versus IP) is an effect modifier: for NEC, death or NDI occurred in 69% after laparotomy versus 85% with drainage (aRR: 0.81; 95% CI: 0.64–1.04).	Although no overall difference in death or NDI, data suggests that, for infants with NEC specifically, initial laparotomy is more likely than initial drainage to reduce death or NDI.
PMID: 39470842 [22] Eaton et al. 2024	STAT trial: stoma or intestinal anastomosis for necrotizing enterocolitis: a multicentre randomized controlled trial	Multi-centre RCT comparing stoma formation (ST) or primary anastomosis (PA) for neonates with NEC requiring intestinal resection. A total of 80 patients randomised. Infants undergoing PA finished PN earlier than ST (HR: 2.38, 95% CI: 1.36–4.12, *p* = 0.004). No difference in mortality or rate of complications requiring unplanned re-operations. Multiple intestinal complications were more frequent in the ST group than the PA group (*p* = 0.02)	If there is no disease distal to resected intestine, primary anastomosis reduces the risk of multiple intestinal complications and does not increase adverse outcomes compared with stoma.
PMID: 41550050 [11] Fernández Ortega et al. 2026	Surgical decision-making strategies in preterm neonates with necrotizing enterocolitis: A randomized controlled clinical trial	Multi-centre RCT evaluating surgical decision-making strategies in NEC, comparing Bell’s criteria to determine surgery (control group—CG) versus combined use of metabolic disorder components (MD7) and paracentesis (intervention group—IG). A total of 117 patients randomised (CG *n* = 56, IG *n* = 61). Mortality was 32.1% in IG and 64.7% in CG (*p* = 0.034; RR = 2, 95% CI: 1.1–4.8). Surgery was performed earlier in the IG group, with better outcomes for perforation, intestinal necrosis, and reoperation (*p* < 0.05).	MD7 and paracentesis was superior to Bell’s criteria for guiding surgical decisions in pre-term NEC, reducing mortality. Due to high sensitivity, some non-therapeutic laparotomies were seen in the IG group.

* Three further publications identified were planned secondary studies or post hoc analyses of the NEST trial. These demonstrated (a) frequent severe growth failure in post-surgical NEC patients, with no difference between drainage and laparotomy, (b) adverse respiratory outcomes with initial drainage compared with primary laparotomy, and (c) good generalisability of the randomised population to the target population [45,53,54].

## Data Availability

No new data were created or analysed in this study. Data sharing is not applicable to this article.

## Abbreviations

The following abbreviations are used in this manuscript:

VLBW/ELBW	Very Low Birth Weight/Extremely Low Birth Weight
SIP	Spontaneous (Focal) Intestinal Perforation
PN	Parenteral Nutrition
NEC	Necrotising Enterocolitis
NICU	Neonatal Intensive Care Unit
CRP	C-Reactive Protein
CGA	Corrected Gestational Age
BAPS-CASS	British Association of Paediatric Surgeons Congenital Anomalies Surveillance System

## Appendix A. Search Strategies

Medline (via Pubmed): (necrotizing enterocolitis[MeSH Terms]) AND (Surgery[MeSH Subheading]) (2000–2026) (RCT only).

Cochrane: MeSH descriptor: [Enterocolitis, Necrotizing] explode all trees and with qualifier(s): [surgery—SU] (2000–2026) (RCT only).

Embase (via Ovid): necrotizing enterocolitis/su and (randomized controlled trial/or controlled clinical trial/) (2000–2026).
